# 

**Published:** 1868-10-15

**Authors:** 


### *CONTENTS:*

PHYSIOLOGY:

Passage of Sanguineous Corpuscles through the walls of the vessels, etc. Translated by Walter Hay, M. D., Associate Editor.... 647
Dysentery. By A. A. Dunn, M. D.... 652

CORRESPONDENCE.... 656

LOOT:

Abortion as a cause of Insanity.... 658
Antiseptic Properties of the Sulphites.... 658
Carbolic Acid and Creasote.... 659
Minute Investigation of the Kidney.... 660
Carbolate of Quinia.... 662
Vesicular Bronchitis of both Lungs in an Infant. Recovery. By David Webster, M. D.... 663
Vaginismus.... 664
Cataract.... 665
Epilepsy.... 666
Thymic Acid.... 668

EDITORIAL:

What's in a Name?.... 668
Rush Medical College.... 670
Medical Imbroglio.... 671
News and Items.... 673

FOREIGN NOTES AND EXCERPTA.... 676

